# *Bordetella pertussis *isolates in Finland: Serotype and fimbrial expression

**DOI:** 10.1186/1471-2180-8-162

**Published:** 2008-09-25

**Authors:** Eriikka Heikkinen, Dorothy K Xing, Rose-Marie Ölander, Jukka Hytönen, Matti K Viljanen, Jussi Mertsola, Qiushui He

**Affiliations:** 1 Pertussis Reference Laboratory, National Public Health Institute, Kiinamyllynkatu 13, 20520 Turku, Finland; 2 National Institute for Biological Standards and Control, Blanche Lane, South Mimms, Potters Bar, Herts, EN6 3QG, UK; 3 Department of Vaccine, National Public Health Institute, Mannerheimintie 166, 00300 Helsinki, Finland; 4 Department of Medical Microbiology and Immunology, University of Turku, Kiinamyllynkatu 13, 20520 Turku, Finland; 5 Department of Pediatrics, Turku University Hospital, Kiinamyllynkatu 4-8, 20520 Turku, Finland

## Abstract

**Background:**

*Bordetella pertussis *causes whooping cough or pertussis in humans. It produces several virulence factors, of which the fimbriae are considered adhesins and elicit immune responses in the host. *B. pertussis *has three distinct serotypes Fim2, Fim3 or Fim2,3. Generally, *B. pertussis *Fim2 strains predominate in unvaccinated populations, whereas Fim3 strains are often isolated in vaccinated populations. In Finland, pertussis vaccination was introduced in 1952. The whole-cell vaccine contained two strains, 18530 (Fim3) since 1962 and strain 1772 (Fim2,3) added in 1976. After that the vaccine has remained the same until 2005 when the whole-cell vaccine was replaced by the acellular vaccine containing pertussis toxin and filamentous hemagglutinin. Our aims were to study serotypes of Finnish *B. pertussis *isolates from 1974 to 2006 in a population with > 90% vaccination coverage and fimbrial expression of the isolates during infection. Serotyping was done by agglutination and serotype-specific antibody responses were determined by blocking ELISA.

**Results:**

Altogether, 1,109 isolates were serotyped. Before 1976, serotype distributions of Fim2, Fim3 and Fim2,3 were 67%, 19% and 10%, respectively. From 1976 to 1998, 94% of the isolates were Fim2 serotype. Since 1999, the frequency of Fim3 strains started to increase and reached 83% during a nationwide epidemic in 2003. A significant increase in level of serum IgG antibodies against purified fimbriae was observed between paired sera of 37 patients. The patients infected by Fim3 strains had antibodies which blocked the binding of monoclonal antibodies to Fim3 but not to Fim2. Moreover, about one third of the Fim2 strain infected patients developed antibodies capable of blocking of binding of both anti-Fim2 and Fim3 monoclonal antibodies.

**Conclusion:**

Despite extensive vaccinations in Finland, *B. pertussis *Fim2 strains were the most common serotype. Emergence of Fim3 strains started in 1999 and coincided with nationwide epidemics. Results of serotype-specific antibody responses suggest that Fim2 strains could express Fim3 during infection, showing a difference in fimbrial expression between *in vivo *and *in vitro*.

## Background

*Bordetella pertussis*, the causative agent of whooping cough or pertussis produces several virulence factors during the course of infection, including adhesins, toxins and lipopolysaccharide [1]. Fimbriae are long, filamentous appendages reaching out from the outer membrane of the bacterium. Structurally they consist of two proteins, major and minor subunit, the latter of which is considered an adhesin [1]. Even though the *in vivo *mechanism by which the fimbriae interact with the host is not fully understood, it is evident that fimbriae elicit protective immune responses and therefore are included in some acellular pertussis vaccines [2-4].

Expression of fimbriae is regulated at two levels: as part of *Bordetella *virulence gene (*bvg*)*-*locus and as an individual gene through phase variation [1,5]. By this form of phenotypic variation where the changes are heritable as well as reversible, bacteria gain increased persistence in the host environment [5]. In *B. pertussis *the level of expression of the two phase-variable major subunits of fimbriae, Fim2 and Fim3, determines the serotype which can therefore be either Fim2, Fim3 or Fim2,3. A homopolymeric tract of cytosine is located at the promoter region of *fim*-genes, and a small deletion or insertion of C's there can cause the phase transition between the high and low level of expression [6]. Serotyping has been an essential part of characterizing *B. pertussis *clinical isolates for a long time [7] and studies on strain variation have shown that *B. pertussis *populations are dynamic and continuously evolving [8,9].

Generally, vaccination has been shown to induce a shift in Fim expression of the strains in a way that in unvaccinated populations Fim2 strains are predominant, while they are largely displaced by Fim3 strains when vaccination is introduced with a whole cell vaccine containing both Fim2 and Fim3 [10-13]. In Finland, whole-cell vaccine has been used since 1952. From 1962 vaccine consisted of the serotype Fim3 strain 18530. Because of the predominance of the Fim2 strains in the country, the serotype Fim2,3 strain 1772 was added. The vaccine with equal amounts of two strains remained the same until 2005 when the whole cell vaccine was replaced by the acellular vaccine containing only pertussis toxin (Ptx) and filamentous hemagglutinin. The vaccine coverage of four doses has been > 90%.

In the present study, we compared serotypes of *B. pertussis *strains circulating in Finland over time. A large number of clinical isolates from 1974 to 2006 were available for the analysis. We also investigated the antibody responses to *B. pertussis *fimbrial antigens during infection and serotype-specific antibody responses by a newly developed blocking ELISA.

## Results

### Serotype of *B. pertussis *isolates

**Figure 1:**
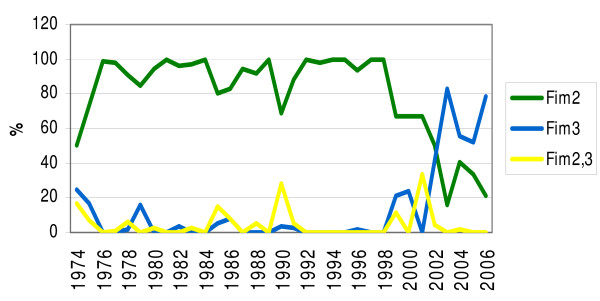
**Serotype distributions of *B. pertussis *isolates in Finland from 1974 to 2006**. A total of 1,109 isolates were collected and serotyped.

From 1974 to 2006, 1,109 isolates were collected and serotyped (Figure 1). In 1974 and 1975, serotype distributions of Fim2, Fim3 and Fim2,3 were 67% (number of Fim2/total number, 28/42), 19% (8/42) and 10% (4/42), respectively. From 1976 to 1998, 94% (667/710) of the isolates were Fim2. Two nationwide epidemics occurred in Finland in 1999 and 2003–2004. In 1999, the frequency of Fim3 started to increase and reached 83% (77/93) in 2003, the highest observed during the study period. The frequency of Fim2,3 strains has remained low throughout the whole period (Figure 1).

### Anti-pertussis IgG antibodies

Serum samples from 41 patients with culture-confirmed pertussis were available for studying of antibody responses to Ptx and purified Fim2/3 mixture. There were 37 paired and 4 single sera. Serotype distributions of the corresponding isolates were 85% for Fim2, 10% for Fim3, and 5% for Fim2,3.

From 37 patients for whom diagnosis was confirmed by culture, paired serum samples were available. Most of the paired serum samples were taken at the early (defined as < 3 weeks of cough) and at the late stage (defined as ≥ 3 weeks of cough) of infection. The mean of interval between the paired sera was 32 days (range, 12–56 days). The levels of anti-Fim and anti-Ptx-IgG antibodies were determined. A significant increase between the paired sera was found in the levels of IgG antibodies to Ptx [28.7 ± 29.3 ELISA units (EU) vs 53.1 ± 22.5 EU] and Fim2/3 mixture (30.4 ± 23.4 EU vs 75.0 ± 18.9 EU) (p < 0.001 for both antigens). For the 4 single sera, IgG antibodies to Ptx were 19.7, 121.7, 64,3 and 9.1 EU and IgG antibodies to Fim2/3 mixture 72,2, 62,7, 37.0 and 28.7 EU.

IgG antibodies were also determined in 60 single serum samples of patients with serologically diagnosed pertussis in 2004. The mean level of IgG antibodies was 79,1 (CV% = 21) for Ptx and 66,0 (CV% = 30,6) for Fim2/3, a level similar to that obtained from second serum samples of patients with culture-confirmed pertussis.

As a negative control, paired serum samples from 10 randomly selected vaccine recipients who received one booster dose of a three-component acellular pertussis vaccine not including fimbriae were analyzed. No change in the levels of anti-Fim2/3 IgG antibodies was found between the paired sera, whereas a significant increase in anti-Ptx IgG levels were observed 30 days after the vaccination (p < 0.005).

### Detection of epitope-specific antibody responses to fimbriae

The blocking assay was developed for the detection of epitope-specific antibodies against the fimbriae. Because there are no purified Fim 2 and Fim3 antigens available separately, it is not possible to differentiate antibodies to Fim2 from those to Fim3 by conventional ELISA. In this blocking assay antibodies formed against Fim2 or Fim3 are detected as they block the binding of monoclonal antibodies to Fim2 or Fim3 antigen, which in this case are Fim2 and Fim3 expressing *B. pertussis *strains. The blocking assay has been previously used for the detection of antibodies against pertactin [14].

First we studied the specific binding of the monoclonal antibodies, designated mAbFim2 and mAbFim3 to the two types of coating antigens: purified Fim2/3 mixture and Fim2- or Fim3-expressing *B. pertussis *strains S1(Fim2) and S3(Fim3). Specific binding of monoclonal antibodies to Fim2- or Fim3-expressing *B. pertussis *strains as well as to Fim2/3 mixture was observed.

Next we examined if there was difference in the blocking of binding of mAbFim2 and mAbFim3 to the two types of coating antigens by human antibodies. Because the blocking of binding of mAbFim2 and mAbFim3 to coating antigens was only observed in presence of serum anti-Fim antibodies and the blocking effect seemed to be dose-dependent (see below), ten serum samples were randomly selected from the serum samples of 41 patients with culture-confirmed pertussis and tested. Although the blocking of binding of mAb to coating antigens was observed, no difference was found in the level of blocking of binding of mAbFim2 and mAbFim3 to the two types of coating antigens (data not shown). The Fim2- and Fim3-expressing strains were eventually chosen as coating antigens for the study because of limited amount of purified Fim2/3 mixture.

**Figure 2:**
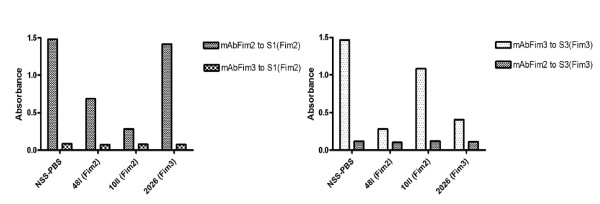
**Blocking of binding of mAbFim2 and mAbFim3 to Fim2-expressing strain S1 (left) and of mAbFim3 and mAbFim2 to Fim3-expressing strain S3 (right) by human IgG antibodies to fimbriae**. Patients 48I and 10II were infected by Fim2 strains and patient 2026 by Fim3 strain. Blocking of binding of mAbFim2 and mAbFim3 to S1 and S3 by normal sheep serum in PBS (NSS-PBS) was not observed. Data were from a single experiment. Three sera with different blocking levels were tested twice and the results obtained were same. In each experiment, both positive and negative controls were included, and all the experiments were performed by an experienced technician.

In presence of serum from patient infected with Fim2 strains, blocking of binding of mAbFim2 to S1(Fim2) was observed (Figure 2, left panel), whereas blocking of binding of mAbFim2 to S1(Fim2) was not changed in presence of serum from patient infected with Fim3 strains. In Figure 2, patients 48I and 10II were infected by Fim2 strains and patient 2026 by Fim3 strain. The level of blocking of binding of mAbFim2 to S1(Fim2) was 54%, 81% and 6%, respectively (Figure 2, left panel). Blocking of binding of mAbFim2 and mAbFim3 to S1(Fim2) and S3(Fim3) by normal sheep serum in PBS (NSS-PBS) was not observed.

In presence of serum from patient infected with Fim3 strain, blocking of binding of mAbFim3 to S3(Fim3) was observed (Figure 2, right panel). However, in presence of sera from some of patients infected with Fim2 strains, blocking of binding of mAbFim3 to S3(Fim3) was also found, indicating the presence of anti-Fim3 antibodies in the patients infected with Fim2 strains. The level of blocking of binding of mAbFim3 to S3(Fim3) was 81%, 26% and 74% for patients 48I, 10II and 2026, respectively (Figure 2, left panel).

No cross-reaction between mAbFim3 and S1(Fim2) or mAbFim2 and S3(FIm3) was observed, no matter in presence or absence of patient serum (Figure 2). Two dilutions (1:10 and 1:100) of serum samples were tested. Blocking of binding of mAbs to Fim2 or Fim3 expressing strains was increased relative to increasing serum concentration, showing that the blocking effect is dose-dependent.

#### Blocking levels of sera from patients with culture-confirmed pertussis

Altogether 41 serum samples from culture-confirmed patients of which 37 had paired samples were investigated for development of specific antibodies against Fim2 and Fim3 during infection. Of the 37 patients with paired sera, 34 were infected by Fim2 strains, two by Fim2,3 strains and one by Fim3 strain. In addition, three single serum samples were obtained from Fim3 infected patients.

All four patients infected with Fim3 strains had high levels of blocking of binding of mAbFim3 to S3(Fim3) (range, 66% to 74% and median, 70%), whereas the blocking levels of mAbFim2 to S1(Fim2) were marginal (range, 3% to 15% and median 8%). Two patients infected with Fim2,3 strains had high levels of blocking of binding of both antibodies, 57% and 56% for mAbFim2 to S1(Fim2) and 80% and 89% for mAbFim3 to S3(Fim3) in their second sera.

The range of serum blocking level of mAbFim2 to S1(Fim2) in the first sera of Fim2-infected patients was from 0% to 62% (median, 11%) and in second sera from 9% to 93% (median, 51%). The difference between paired sera was statistically significant (p < 0.0001). The blocking levels of mAbFim3 to S3(Fim3) were also tested for Fim2-infected patients. Thirty-four Fim2-infected patients were divided into three groups according to level of IgG antibodies to Fim2/3 mixture and blocking levels of mAbFim2 to S1(Fim2) and mAbFim3 to Fim3(S3) between the paired sera.

**Figure 3:**
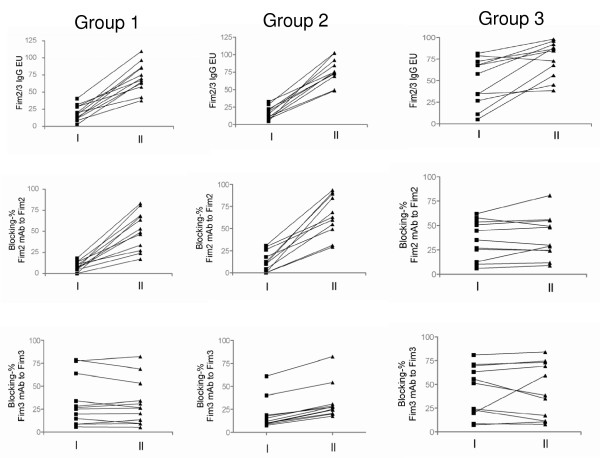
**Detection of epitope-specific antibody responses to fimbriae in paired sera from Fim2 infected patients**. Group 1 included 12 patients who had significant increases in level of IgG antibodies to fimbriae and in blocking level of mAbFim2 to S1(Fim2) between paired sera (p < 0.01) but had no change in blocking level of mAbFim3 to S3. Group 2 consisted of 11 patients who had significant increases in level of IgG antibodies to fimbriae and in blocking levels of both mAbFim2 to S1(Fim2) and mAbFim3 to S3(Fim3) between paired sera (p < 0.01 for both). Group 3 included 11 patients who had no change either in level of IgG antibodies to fimbriae or in blocking levels of both mAbFim2 to S1(Fim2) and mAbFim3 to S3(Fim3) between paired sera.

Group 1 included 12 patients who had significant increases in level of IgG antibodies to Fim2/3 mixture and in blocking level of mAbFim2 to S1(Fim2) between paired sera (p < 0.01) but had no change in blocking level of mAbFim3 to S3(Fim3) (Figure 3).

Group 2 consisted of 11 patients who had significant increases in level of IgG antibodies to Fim2/3 mixture and in blocking levels of both mAbFim2 to S1(Fim2) and mAbFim3 to S3(Fim3) between paired sera (p < 0.01 for both) (Figure 3).

Group 3 included 11 patients who had no change either in level of IgG antibodies to Fim2/3 mixture or in blocking levels of both mAbFim2 to S1(Fim2) and mAbFim3 to S3(Fim3) between paired sera (Figure 3).

Of the 34 Fim2-infected patients, 11 patients had already had high level of the blocking (≥ 40%) of mAbFim3 to S3(Fim3) in the first sera, and in 9 of them the blocking level remained high in the second sera. Of the 11 patients, 3 were in group 1, 2 in group 2, and 6 in group 3 (Figure 3). Taken together, the patients who were infected with Fim2 strains, can also produce anti-Fim3 antibodies which indicate that Fim2 strains may express Fim3 during the course of infection.

#### Blocking levels of sera from patients with serologically confirmed pertussis

Of the 34 patients who were infected by Fim2 strains and had paired sera (see above), 23 (Groups 1 and 2) had significant increases in level of IgG antibodies to Fim2/3 mixture and in blocking level of mAbFim2 to S1(Fim2) (Figure 3). All the 23 patients had blocking level of mAbFim2 to S1(Fim2) ≤ 30% in their first sera (Figure 3). Of the four patients infected by Fim3 strains, one had paired sera and three had only single sera. All four patients had blocking level of binding of mAbFim3 to S3(Fim3) > 50%. Therefore, 40% was empirically determined as cut-off level and considered significant.

In 60 sera of patients with serologically diagnosed pertussis in 2004, the range of blocking level was 0% to 86.4% (median, 23.1%) for mAbFim2 to S1(Fim2) and 7.4% to 91.7% (median, 63.9%) for mAbFim3 to S3(Fim3). In 42 sera the blocking level of mAbFim3 to S3(Fim3) was considered significant (≥ 40%), whereas in 16 sera the blocking level of mAbFim2 to S1(Fim2) was significant. In five sera the significant blocking levels were detected for both mAbFim2 to S1(Fim2) and mAbFim3 to S3(Fim3).

Since no blocking of mAbFim2 to S1(Fim2) was detected in sera of culture-confirmed patients infected by Fim3 strains (see above section), the five patients who had significant blocking for both seemed to be infected by Fim2 or Fim2,3 strains. If this is the case, 37 (62%) of 60 patients would have been infected by Fim3 strains. In Finland, a nationwide epidemic occurred in 2003 to 2004. During this period, the serotype distributions of Fim2, Fim3 and Fim2,3 were 32%, 66% and 1%, respectively, when 231 *B. pertussis *isolates were collected and tested.

## Discussion

In this study, we present the epidemiology of different serotypes of *B. pertussis *strains circulating in Finland where the vaccination against pertussis has been used since early 1950s. Results are based on an analysis of a large number of isolates collected over time covering four decades. In addition, we investigated human antibody responses to *B. pertussis *fimbrial antigens during infection and serotype-specific antibodies by a newly developed blocking ELISA. Our data suggest that Fim2 strains can express both Fim2 and Fim3 during infection, showing a difference in fimbrial expression between *in vitro *and *in vivo*. To our knowledge, this is the first study that shows that *B. pertussis *serotype detected *in vitro *may not reflect its expression *in vivo*.

Many studies have shown that Fim2 isolates predominate in unvaccinated populations, while they are largely displaced by Fim3 strains when vaccination is introduced with a whole cell vaccine containing both Fim2 and Fim3 [10-13]. In Sweden, before 1979 when the whole-cell vaccine was used, 70% of circulating strains were Fim3 [11]. From 1979 to 1995 when the pertussis vaccination was stopped, Fim2 started to increase and reached 64% in early 1990s. In 1996 when general vaccination with an acellular vaccine was reintroduced, the prevalence of Fim2 declined and Fim3 strains emerged rapidly. In 2002 and 2003, serotype Fim3 constituted 96% among fully vaccinated individuals. In contrast to the high prevalence of Fim3 strains in many countries with long-term vaccination, Fim2 has been the prevalent serotype in Finland for more than 20 years. The Finnish whole cell vaccine contains two strains expressing Fim3 and Fim2,3, and this vaccine has remained the same since 1976. The exact reasons for the dominance of serotype Fim2 strains in Finland are not known. One explanation is that different whole cell vaccines were used in different countries. The exact amounts of Fim2 and Fim3 in these vaccines are unknown, although all whole cell pertussis vaccines used in the world contain both Fim2 and Fim3 as recommended by WHO. Another explanation is that immunity to Fim2 and Fim3 in Finnish population might not be the same as in other populations. The population immunity to fimbrial antigens comes from vaccination or natural infection or both. We have previously compared clinical isolates recovered since the early 1990s in Finland and France, two countries with similar histories of long-term mass vaccination with whole cell vaccines. In France, 90% of isolates in 1990s were Fim3. Furthermore, although isolates in both countries were genetically similar, they varied temporally [15].

It is recommended that monoclonal antibodies are used for serotyping of *B. pertussis *and that Fim2- and Fim3-expressing reference strains are included as controls [10-12]. The two mAbs used in the present study were produced from hybridoma cell lines BPF2 and BPC10 and were proven to be specific for serotype Fim2 and serotype Fim3 [16]. They are being used in many countries for serotyping. The same mAbs and reference strains were also used in the blocking ELISA. No cross reaction was found between mAbs and reference strains used as coating antigens. Moreover, all patients who were infected with Fim3 strains had developed specific antibodies to Fim3 but not to Fim2. The development of anti-Fim3 antibodies was also observed in sera of patients with serologically diagnosed pertussis in 2004 when Fim3 strains were prevalent.

It is known that *B. parapertussis *also produces similar virulence factors including fimbriae. In Finland, infections caused by *B. parapertussis *are detected in children 2 to 8 years of age [17]. Eight Finnish *B. parapertussis *strains isolated during 1990s were randomly selected from our strain collection and tested for agglutination with the two mAbs used in the present study and no agglutination was detected (data not shown). This indicates that there is no cross reaction between the mAbs and *B. parapertussis*. However, it remains to be studied if sera from parapertussis patients can block the binding of the mAbs to Fim2- or Fim3-expressing strains.

Fim 2 and 3 are similar in structure since they have an NH_2_-terminal amino acid sequence homology of about 75% and have identical helical structures demonstrated by electron microscopic analysis [18-20]. Moreover, the two fimbriae were found to contain unique antigenic epitopes, which is consistent with their being serotype-specific agglutinogens [16]. In a previous report, Robinson and his colleagues showed that Fim2 and Fim3 are generally not cross-reactive [21]. Although some cross-reactivity was mentioned, the cross-reactive epitopes could be non-protective epitopes. They also found that immunization of mice with sero-specific agglutinogen resulted in immune selection so that organisms recovered following infection did not express the immunizing antigen [21]. In this present study, 32% of patients who were infected with Fim2 strains had developed IgG antibodies which blocked the binding of anti-Fim3 monoclonal antibodies to Fim3, indicating that the antibodies were induced by Fim3 antigen. Therefore, in addition to expression of Fim2, the Fim2 strains might express Fim3 *in vivo *during the interaction with host cells. Alternatively, these patients might have been previously infected with a Fim3 strain. Upon subsequent infection with a Fim2 strain, not only a new immune response to Fim2 specific epitopes but also a boosting response to Fim2/3 cross-reactive epitopes (that would be very close to those bound by the mAbs) would be induced. It is interesting to study if there is cross-reactive immune response induced by the two fimbriae and if the cross-reactive immune response is protective or not. The expression of different fimbrial major subunits is phase variable and is a consequence of insertion or deletion of cytosines in C-rich region at the promoter region of *fim2 *and *fim3 *genes [6]. Phase variation is one means for the bacterium to create phenotypic divergence and to avoid host defense mechanisms [5]. Fimbriae have also shown some level of phase variation *in vivo *[6,22]. *Neisseria meningitidis *has a phase-variable immunogenic outer membrane protein, PorA, which has a control mechanism similar to *Bordetella *fimbriae. The length of variable homopolymeric tracts at the promoter regions of PorA showed variation between strains isolated from carriers and strains isolated from patients [23].

Recently, the annotated genome sequence of *B. pertussis *was published [24]. The genome contains 3,816 ORFs. According to the sequenced genome, the genes encoding Fim2 (BP1119) and Fim3 (BP1568) are located apart from each other, suggesting that in certain circumstances the regulation of expression of the two genes may be different. A recent study where microarray-based gene expression profiling was used, reported differential modulation of *B. pertussis *virulence genes [25]. In the *B. pertussis *strain, which represents serotype Fim2, the *fim3 *gene was found to be expressed in virulence repressing conditions, whereas *fim2 *was expressed in virulence activating conditions and confirmed to be a virulence activated gene. Another study has also confirmed that in *B. pertussis fim2 *gene but not *fim3 *gene was *Bvg*-activated gene [26].

In vaccinated populations, symptoms of pertussis can be mild and the patients do not usually seek for medical help. Therefore the possibility that some of patients who were infected by Fim2 strains may have already had infections caused by Fim3 strains, could not be excluded. However, the frequency of circulating Fim3 strains was very low in Finland during the period when the sera were collected. Also, according to the Finnish vaccination schedule and the age of patients at the time of sampling, it is unlikely that the anti-Fim3 antibodies detected in patients who were infected by Fim2 strains were due to recent vaccinations.

In the present study, some patients who were infected by Fim2 strains had already had anti-Fim3 antibodies in the first sera (Figure 3), and in most of them levels of the antibodies remained unchanged in the second sera. One explanation might be that the first serum samples had been taken when the patients were already at the convalescent phase or these patients had developed anti-Fim antibodies exceptionally rapidly.

*B. pertussis *continues to cause epidemics in vaccinated populations in spite of high vaccination coverage. As a result of this study we gained more information of the possible mechanisms the bacterium uses to infect highly vaccinated populations. This information is important for the future development of vaccines against pertussis.

## Conclusion

Despite extensive vaccinations in Finland, Fim2 strains were the most common serotype. Fim3 strains emerged since 1999 and the emergence coincided with nationwide epidemics. In a population with long-term vaccinations, Fim2 strains could express Fim3 during infection, showing a difference in fimbrial expression between *in vivo *and *in vitro*.

## Materials and methods

### Bacterial strains, serum samples and study subjects

A total of 1,109 *B. pertussis *isolates were obtained from 1974 to 2006 and stored in strain collections of the Department of Vaccine, National Public Health Institute, Helsinki (from 1974 to 1994) and the Pertussis Reference Laboratory, National Public Health Institute, Turku (from 1991 on). The number of isolates collected each year varied from 4 to 138 and was 112 in 1974–1976, 182 in 1977–1979, 70 in 1980–1982, 70 in 1983–1985, 67 in 1986–1988, 81 in 1989–1991, 78 in 1992–1994, 78 in 1995–1997, 77 in 1998–2000, 121 in 2001–2003, 173 in 2004–2006. The isolates had been sent from the local clinical microbiology laboratories or collected during the outbreaks of pertussis.

Of the strains studied, 41 were isolated from patients from whom serum samples were also available. The serum samples were collected at the Pertussis Reference Laboratory of the National Public Health Institute, Turku, Finland. No selection of the serum samples was done, because all the patients from whom both sera and bacterial strains were available were included. Of the 41 patients, 39 were diagnosed in 1990s [27,28], one was in 2004 and one was in 2005. Of the 23 patients whose vaccination status was investigated, 19 had received four doses of Finnish diphtheria-tetanus-whole cell pertussis vaccine and 4 had received three doses of the same vaccine. None of the 41 patients had received acellular pertussis vaccine because in Finland the acellular vaccine was introduced in January 2005. Age of the 41 patients ranged from 4 months to 64 years (mean, 17 years and median, 10 years). Of the patients, 37 had paired sera and 4 single sera. The mean of interval between the paired sera was 32 days (range, 12–56 days). Of the 37 patients who had paired sera, 32 had already cough at the first blood sampling, three developed cough later and two remained asymptomatic [27,28].

Fim2 strains predominated in 1990s and Fim3 strains started to increase since 1999. Because only two culture-positive patients had sera available since 2000, 60 sera from patients who were serologically diagnosed in 2004 at the Department of Medical Microbiology and Immunology, University of Turku, Turku were included [29]. The selection criteria for the 60 patients were based on diagnostic level of serum anti-Ptx IgG antibodies. Age of the patients ranged from 1 to 47 years (mean, 17 years). Only single sera were available from the patients.

In addition, paired serum samples from 10 vaccinees were randomly selected to serve as negative controls for detection of antibody responses to fimbrial antigens. The vaccinees received one booster dose of acellular pertussis vaccine containing Ptx, filamentous hemagglutinin and pertactin but not fimbriae [30]. The serum samples were taken before and 30 days after the booster vaccination. Age of the subjects ranged from 10 to 13 years.

### Serotyping

Serotyping of *B. pertussis *strains was done by slide agglutination or indirect ELISA. The slide agglutination test was performed as described previously [31]. In the indirect ELISA method, validated by Tsang et al. [32] the microtiter plate was coated overnight at room temperature with 100 μl of *B. pertussis *suspension, inactivated at 56°C for 1 hour, with OD 0,1 at 620 nm. Strains specifically expressing either Fim2(S1) or Fim3(S3), kindly provided by Dr. D. Xing, National Institute for Biological Standards and Control (NIBSC), UK, were included in each run as positive controls. Wells were washed with phosphate-buffered saline (PBS) and blocked with 150 μl 1% normal sheep serum (NSS) (Autogen Bioclear UK Ltd, Wiltshire, UK) in PBS (NSS-PBS) at 37°C for 1 hour. After the blocking and each following incubation step wells were washed three times with 0,05% (w/v) NaCl, 0,05%Tween20 (Sigma, St.Louis, USA) (v/v). Anti-Fim2 or anti-Fim3 monoclonal antibodies (mAbFim2 or mAbFim3) diluted in PBS (1:1000), produced and provided by NIBSC, UK were added, and the plate was incubated at 37°C for 2 hours. Anti-mouse antibodies (DakoCytomation, Glostrup, Denmark) (1:2000 in NSS-PBS) were added, and the plate was incubated at 37°C for 2 h. After the addition of phosphatase substrate (Sigma, Steinheim, Germany) in diethanolamine-MgCl_2_-buffer (Reagena, Toivala, Finland), absorbance at 405 nm was measured with Victor 1420 Multilabel counter (Wallac, Turku, Finland) consecutively until the absorbance of control strains reached 1,5.

### ELISA for determination of serum IgG antibody

For determination of serum IgG antibodies to Ptx and purified Fim2/3 mixture microtiter plates were coated with 1 μg/ml of purified Ptx or purified Fim2/3 mixture for overnight at room temperature. Ptx was kindly provided by GlaxoSmithKline Biologicals, Rixensart, Belgium. Fim2/3 mixture preparation (code 85/549) was provided by NIBSC, UK and with purity of ≥ 95% of Fim 2/3 components by SDS-PAGE. Due to absence of standards for purified Fim 2 and Fim 3 antigen, the proportion of each Fim2 and Fim3 in this mixture preparation has not been defined. If assuming similar affinity for both anti-Fim2 and Fim3 mAbs to the antigens, this preparation contains 10% higher amount of Fim 2 than Fim 3 by in-house ELISAs. The detailed ELISA used in the present study was described earlier [14,27]. Plates were washed with 200 μl PBS and then incubated with 150 μl 1% NSS-PBS at 37°C for 1 hour. Serum samples diluted 1:100 were added and incubated in duplicate wells at 37°C for 2 hours. After IgG conjugate diluted 1:800 (DakoCytomation) was incubated at 37°C for 2 hours, 100 μl of phosphatase substrate (Sigma) in diethanolamine-MgCl_2_-buffer (Reagena) was added. Absorbance in 405 nm was measured consecutively until the absorbance of positive serum pool reached 1,5. The positive and negative pool sera were included in each run, and results were expressed as relative ELISA units (1 ELISA unit, 1:100 of the corresponding antibody concentration in the positive pool sera).

### Blocking ELISA

Blocking ELISA [14] was used to test the ability of human antibodies to compete with specific monoclonal antibodies in binding to Fim2 or Fim3 expressing strains. The mAbs used in blocking ELISA were the same as those for serotyping. Microtiter plates were coated with 100 μl of bacterial suspension of *B. pertussis *strains expressing either Fim2 or Fim3 for overnight at room temperature (see section of Serotyping). Plates were washed with 200 μl PBS, pH 7, 2–7, 4 and incubated with 150 μl 1% NSS-PBS at 37°C for 1 hour and washed three times with 200 μl 0,05% NaCl, 0,05% Tween20 (Sigma). The NaCl-Tween buffer was used for washings. One hundred microliter of serum samples diluted 1:10 were added in duplicate wells and were incubated at 37°C for 2 hours. NSS-PBS and samples from negative and positive serum pool were used as controls and included in each run. One hundred microliter of mAbFim2 or mAbFim3 diluted 1:1000 in PBS were added, and the plates were incubated at 37°C for 2 hours. Anti-mouse conjugate (DakoCytomation) diluted 1:2000 in NSS-PBS was added and incubated at 37°C for 2 hours. Finally, 100 μl of phospathase substrate (Sigma) in diethanolamine-MgCl_2_-buffer (Reagena) was added. Absorbance in 405 nm was measured consecutively until the absorbance of NSS-PBS was reached to 1,5. The relative blocking of binding of mAb to Fim2 or Fim3 was calculated as 100% – absorbance of testing serum/absorbance of NSS-PBS. Blocking level of a serum ≥ 40% was considered significant.

### Statistical analysis

Paired t-test was used for statistical analysis of the differences in level of antibodies and relative blocking of binding of mAb between paired serum samples. P value < 0,05 was considered significant.

## Authors' contributions

QH conceived and designed the experiments. EH, JM and QH analyzed the data. R-MÖ and QH guided to serotyping. JH and MKV guided to routine diagnosis of pertussis. DKX provided monoclonal antibodies and reference strains. EH and QH wrote the manuscript with helpful discussions and comments from all co-authors. All authors read and approved the final manuscript.

## References

[B1] Smith AM, Guzman CA, Walker MJ (2001). The virulence factors of Bordetella pertussis: a matter of control. FEMS Microbiol Rev.

[B2] Mattoo S, Miller JF, Cotter PA (2000). Role of Bordetella bronchiseptica Fimbriae in Tracheal Colonization and Development of a Humoral Immune Response. Infect Immun.

[B3] Rodríguez ME, Hellwig SM, Pérez Vidakovics ML, Berbers GA, Winkel JG van de (2006). Bordetella pertussis attachment to respiratory epithelial cells can be impaired by fimbriae-specific antibodies. FEMS Immunol Med Microbiol.

[B4] Olin P, Rasmussen F, Gustafsson L, Hallander HO, Heijbel H (1997). Randomised controlled trial of two-component, three-component, and five-component acellular pertussis vaccines compared with whole-cell pertussis vaccine. Lancet.

[B5] Woude MW van der, Baumler AJ (2004). Phase and antigenic variation in bacteria. Clin Microbiol Rev.

[B6] Willems R, Paul A, Heide HG van der, ter Avest AR, Mooi F (1990). Fimbrial phase variation in Bordetella pertussis: a novel mechanism for transcriptional regulation. EMBO J.

[B7] Eldering G, Hornbeck C, Baker J (1957). Serological study of Bordetella pertussis and related species. J Bacteriol.

[B8] Heikkinen E, Kallonen T, Saarinen L, Sara R, King A, Mooi F, Soini J, Mertsola J, He Q (2007). Comparative genomics of Bordetella pertussis reveals progressive gene loss in Finnish strains. PLoS ONE.

[B9] van Amersfoorth SCM, Schouls LM, Heide HGJ van der, Advani A, Hallander HO, Bondeson K, von König CHW, Riffelmann M, Vahrenholz C, Guiso N, Caro V, Njamkepo E, He Q, Mertsola J, Mooi FR (2005). Analysis of Bordetella pertussis populations in European countries with different vaccination policies. J Clin Microbiol.

[B10] Preston NW, Carter EJ (1992). Serotype specificity of vaccine-induced immunity to pertussis. Commun Dis Rep CDR Rev.

[B11] Hallander HO, Advani A, Donnelly D, Gustafsson L, Carlsson R-M (2003). Shifts of Bordetella pertussis variants in Sweden from 1970 to during three periods marked by different vaccination programs. J Clin Microbiol.

[B12] Borisova O, Kombarova SY, Zakharova NS, van Gent M, Aleshkin VA, Mazurova I, Mooi FR (2007). Antigenic divergence between Bordetella pertussis clinical isolates from Moscow, Russia, and vaccine strains. Clin Vaccine Immunol.

[B13] van Loo IHM, Mooi FR (2002). Changes in the Dutch Bordetella pertussis population in the first 20 years after the introduction of whole-cell vaccines. Microbiology.

[B14] He Q, Mäkinen J, Berbers G, Mooi FR, Viljanen MK, Arvilommi H, Mertsola J (2003). Bordetella pertussis protein pertactin induces type-specific antibodies: one possible explanation for the emergence of antigenic variants?. J Infect Dis.

[B15] Caro V, Elomaa A, Brun D, Mertsola J, He Q, Guiso N (2006). Bordetella pertussis, Finland and France. Emerg Infect Dis.

[B16] Li ZM, Brennan MJ, David JL, Carter PH, Cowell JL, Manclark CR (1988). Comparison of type 2 and type 6 fimbriae of Bordetella pertussis by using agglutinating monoclonal antibodies. Infect Immun.

[B17] He Q, Viljanen M, Arvilommi H, Aittanen B, Mertsola J (1998). Whooping cough caused by Bordetella pertussis and Bordetella parapertussis in an immunized population. JAMA.

[B18] Cowell JL, Zhang JM, Urisu A, Suzuki A, Steven AC, Liu T, Liu TY, Manclark CR (1987). Purification and characterization of serotype 6 fimbriae from Bordetella pertussis and comparison of their properties with serotype 2 fimbriae. Infect Immun.

[B19] Mooi FR, Heide HG van der, ter Avest AR, Welinder KG, Livey I, Zeijst BA van der, Gaastra W (1987). Characterization of fimbrial subunits from Bordetella species. Microb Pathog.

[B20] Steven AC, Bisher ME, Trus BL, Thomas D, Zhang JM, Cowell JL (1986). Helical structure of Bordetella pertussis fimbriae. J Bacteriol.

[B21] Robinson A, Corrings AR, Funnell SG, Fernandez M (1989). Serospecific protection of mice against intranasal infection with Bordetella pertussis. Vaccine.

[B22] Preston NW, Timewell RM, Carter EJ (1980). Experimental pertussis infection in the rabbit: Similarities with infection in primates. J Infect.

[B23] Ende A van der, Hopman CTP, Dankert J (2000). Multiple mechanisms of phase variation of PorA in Neisseria meningitidis. Infect Immun.

[B24] Parkhill J, Sebaihia M, Preston A, Murphy LD, Thomson N, Harris DE, Holden MTG, Churcher CM, Bentley SD, Mungall KL, Cerdeno-Tarraga AM, Temple L, James K, Harris B, Quail MA, Achtman M, Atkin R, Baker S, Basham D, Bason N, Cherevach I, Chillingworth T, Collins M, Cronin A, Davis P, Doggett J, Feltwell T, Goble A, Hamlin N, Hauser H, Holroyd S, Jagels K, Leather S, Moule S, Norberczak H, O'Neil S, Ormond D, Price C, Rabbinowifsch E, Rutter S, Sanders M, Saunders D, Seeger K, Sharp S, Simmonds M, Skelton J, Squares R, Squares S, Stevens K, Unwin L, Whitehead S, Barrell BG, Maskell DJ (2003). Comparative analysis of the genome sequences of Bordetella pertussis, Bordetella parapertussis and Bordetella bronchiseptica. Nat Genet.

[B25] Hot D, Antoine R, Renauld-Mongenie G, Caro V, Hennuy B, Levillain E, Huot L, Wittmann G, Poncet D, Jacob-Dubuisson F, Guyard C, Rimlinger F, Aujame L, Godfroid E, Guiso N, Quentin-Millet MJ, Lemoine Y, Locht Cl (2003). Differential modulation of Bordetella pertussis virulence genes as evidenced by DNA microarray analysis. Mol Genet Genomics.

[B26] Cummings CA, Bootsma HJ, Relman DA, Miller JF (2006). Species- and strain-specific control of a complex, flexible regulon by Bordetella BvgAS. J Bacteriol.

[B27] He Q, Viljanen M, Nikkari S, Lyytikainen R, Mertsola J (1994). Outcomes of Bordetella pertussis infection in different age groups of an immunized population. J Infect Dis.

[B28] Minh NNT, He Q, Edelman K, Ölander R-M, Viljanen MK, Arvilommi H, Mertsola J (1999). Cell-mediated immune responses to antigens of Bordetella pertussis and protection against pertussis in school children. Pediatr Infect Dis J.

[B29] Viljanen M, Ruuskanen O, Granberg C, Salmi T (1982). Serological diagnosis of pertussis: IgM, IgA and IgG antibodies against Bordetella pertussis measured by enzyme-linked immunosorbent assay (ELISA). Scand J Infect Dis.

[B30] Tran Minh NN, He Q, Ramalho A, Kaufhold A, Viljanen MK, Arvilommi H, Mertsola J (1999). Acellular vaccines containing reduced quantities of pertussis antigens as a booster in adolescents. Pediatrics.

[B31] Elomaa A, Advani A, Donnelly D, Antila M, Mertsola J, Hallander H, He Q (2005). Strain Variation among Bordetella pertussis isolates in Finland, where the whole-cell pertussis vaccine has been used for 50 years. J Clin Microbiol.

[B32] Tsang RSW, Sill ML, Advani A, Xing D, Newland P, Hallander H (2005). Use of monoclonal antibodies to serotype Bordetella pertussis isolates: comparison of results obtained by indirect whole-cell enzyme-linked immunosorbent assay and bacterial microagglutination methods. J Clin Microbiol.

